# Age-related injury responses of human oligodendrocytes to metabolic insults: link to BCL-2 and autophagy pathways

**DOI:** 10.1038/s42003-020-01557-1

**Published:** 2021-01-04

**Authors:** Milton Guilherme Forestieri Fernandes, Julia Xiao Xuan Luo, Qiao-Ling Cui, Kelly Perlman, Florian Pernin, Moein Yaqubi, Jeffery A. Hall, Roy Dudley, Myriam Srour, Charles P. Couturier, Kevin Petrecca, Catherine Larochelle, Luke M. Healy, Jo Anne Stratton, Timothy E. Kennedy, Jack P. Antel

**Affiliations:** 1 Neuroimmunology Unit, Montreal Neurological Institute and Department of Neurology and Neurosurgery, McGill University, 3801 University Street, Montreal, QC H3A 2B4 Canada; 2 Douglas Mental Health University Institute, 6875 Boulevard LaSalle, Verdun, QC H4H 1R3 Canada; 3 Department of Neurosurgery, McGill University Health Centre and Department of Neurology and Neurosurgery, McGill University, 3801 University Street, Montreal, QC H3A 2B4 Canada; 4 Department of Pediatric Neurosurgery, Montreal Children’s Hospital, 1001 Décarie Boulevard, Montreal, QC H4A 3J1 Canada; 5 Division of Pediatric Neurology, Montreal Children’s Hospital, 1001 Décarie Boulevard, Montreal, QC H4A 3J1 Canada; 6 Department of Neurology, University of Montreal, 1051 Saguinet Street, Montreal, QC H2X 3E4 Canada; 7 Department of Neurology and Neurosurgery; Department of Human Genetics and Bioengineering, McGill University, 3801 University Street, Montreal, QC H3A 2B4 Canada

**Keywords:** Multiple sclerosis, Cellular neuroscience, Apoptosis, Autophagy

## Abstract

Myelin destruction and oligodendrocyte (OL) death consequent to metabolic stress is a feature of CNS disorders across the age spectrum. Using cells derived from surgically resected tissue, we demonstrate that young (<age 5) pediatric-aged sample OLs are more resistant to in-vitro metabolic injury than fetal O4+ progenitor cells, but more susceptible to cell death and apoptosis than adult-derived OLs. Pediatric but not adult OLs show measurable levels of TUNEL+ cells, a feature of the fetal cell response. The ratio of anti- vs pro-apoptotic BCL-2 family genes are increased in adult vs pediatric (<age 5) mature OLs and in more mature OL lineage cells. Lysosomal gene expression was increased in adult and pediatric compared to fetal OL lineage cells. Cell death of OLs was increased by inhibiting pro-apoptotic BCL-2 gene and autophagy activity. These distinct age-related injury responses should be considered in designing therapies aimed at reducing myelin injury.

## Abstract (web summary)

Milton Fernandes et al. combine single cell RNA-sequencing and functional assays to show that human oligodendrocytes exhibit an age-dependent resistance to apoptosis. Their findings have important implications for the pathology and treatment of myelination disorders like multiple sclerosis.

## Introduction

Oligodendrocyte (OL) injury with subsequent cell death consequent to metabolic insults is a feature of a number of acquired disorders of the central nervous system (CNS) across the age spectrum. These include adult-onset multiple sclerosis (MS) and focal and diffuse injury in children. In the acute lesions in MS, there is limited loss of OLs^1,2^ with inconsistent evidence of active cell death response mechanisms as measured by presence of OLs with morphologic features of apoptosis or cleaved caspase-3^3,4^. Surviving OLs show disruption of terminal cell processes consistent with a dying-back phenomenon^5,6^, which may reflect sub-lethal injury that is potentially reversible^7,8^ and allow them contribute to subsequent myelin repair^9,10^. In chronic lesions, there is universal loss of OLs^1^. Although immune-mediated mechanisms are considered to underlie initial lesion formation, analysis of MS tissues provides evidence of local ischemia/hypoxia contributing to ongoing injury^11–15^. These responses are attributed to disturbance of the micro-circulation due to focal edema or local production of toxic metabolites that interfere with energy metabolism^16^. Dutta and Trapp, using tissue micro-dissected from established MS lesions, found no evidence of activation of programmed cell death pathways to account for the OL loss^17^. The variable myelin repair in MS is mainly attributed to OPCs present in lesions^18^. Our previous studies suggest that OPCs in MS lesions are even more susceptible to injury than mature OLs^19^.

Across the pediatric age range, metabolic insults can result in permanent motor and cognitive deficits^20^. Underlying causes for such insults include systemic metabolic disorders and infection, trauma, and prolonged seizures^21–24^. Such insults in full term new born infants are referred to as neonatal hypoxic/ischemic encephalopathy (reviewed in 21). The pathologic features include involvement of subcortical and central white matter similar to what is observed in periventricular leukomalacia that occurs in pre-term infants^20,25^. Pre-myelinating oligodendrocytes; phenotypically characterized as O4+/platelet derived growth factor-receptor α (PDGF-R+)/NG2+ progenitor cells, remain abundant after birth^26–28^ and are particularly vulnerable to this form of injury^29–31^. Most injury was considered to be via necrosis although some degree of apoptosis could be observed^32^. We identified such a cell population in our previous single cell RNA sequencing analysis of surgically derived pediatric age brain samples^33^. Cell death of OPCs was shown to occur by apoptosis in an in vivo neonatal rat model of hypoxic-ischemic injury^34,35^.

In our current study we demonstrate the age-related differences in susceptibility of human OL lineage cells to metabolic injury and relate these to the underlying molecular mechanisms, specifically the interaction of BCL-2 family molecules that regulate the intrinsic apoptotic pathway^36^ (illustrated in Supplementary Fig. 1) and the autophagy pathway. The BCL-2 family is divided in three groups: the pro-apoptotic effector molecules BAX and BAK, the anti-apoptotic BCL-2, BCL-XL (encoded by *BCL2L1* gene), BCL-2A1, MCL-1, BCL-W (encoded by *BCL2L2* gene) and BCL-B (encoded by *BCL2L10* gene) and the sensor/activator molecules from the BH3-only subfamily (BAD, BIM, BID and others)^37–39^. Under stress, BH3-only subfamily members are activated or upregulated. They can bind to the anti-apoptotic family members, preventing their interaction with BAX and BAK, or bind directly to BAX and BAK to result in their release from inhibition, triggering the apoptotic pathway. The BCL-2: BAX ratio has been used as a measure of the relative expression of anti- vs pro-apoptotic molecules^37^. In vitro studies indicate that pro-apoptotic family members are constitutively expressed at a considerably higher level than anti-apoptotic family members in rat OPCs^40,41^. With differentiation, the expression of anti- vs pro-apoptotic members increases^40–42^, potentially increasing resistance to apoptosis. We provide evidence for the significance of the BCL-2 pathway in protecting human OLs.

Autophagy reflects a metabolic switch from anabolism to catabolism with degraded cellular components being used as a source of energy. This process initially supports the survival of the cell under nutrient starvation^43,44^. However, the formation of autophagosomes without a further fusion with lysosomes can be detrimental to the cell, ultimately leading to cell death (autophagic cell death); such cell death can occur either dependently or independently of apoptosis^43–52^. In a neonatal mouse model of asphyxia, OL death was increased by preventing autophagy^53,54^. Neuman et al. found that fasting or treatment with metformin could reverse age-related decreases in metabolic function and protect against DNA damage in aged rat A2B5+ OPCs, resulting in enhanced myelination capacity^55,56^. In a previous study we had observed that metabolic stress conditions induced an enhanced autophagy response in adult human OLs as measured by increased expression of LC3^8^. We now address the role of autophagy in regulating OL cell death in response to metabolic insult.

For our study, we isolated OLs from surgical resections of pediatric and adult cases to (i) determine their relative susceptibility to metabolic insult (LG/NG) conditions in cell culture assays and to (ii) identify molecular signatures related to cell death and potential protective pathways linked with the observed functional responses based on whole-cell single cell RNA sequencing (scRNAseq). Comparisons are also made with OL lineage cells derived from second trimester fetal brain samples. Our findings show that pediatric age OLs have acquired resistance to BCL-2 family apoptotic mediated injury compared to fetal OPCs but residual susceptibility persists relative to corresponding cells present in the adult CNS. We also now show that genes responsible for the formation of lysosomes are upregulated in pediatric and adult OLs ex vivo compared to fetal O4 + cells and use in vitro blocking assays to indicate the initial protective effect of the autophagy response induced by LG conditions.

## Results

### Functional studies demonstrate age-related differences in injury responses of human OLs to metabolic stress

To model conditions of metabolic stress in vitro and to investigate whether there is an age-related difference in the protective response to cell death, we utilized dissociated cultures of OLs derived from adult and pediatric surgical samples and O4+ cells derived from fetal samples. We compared relative levels and underlying mechanisms of cell death between cells cultured in optimal conditions (DMEM/F12 + N1, hereafter referred to as N1) and cells cultured under sub-optimal conditions i.e. reduced overall nutrients (DMEM alone) plus low and no glucose (LG/NG) conditions. We documented that the cultures from the pediatric donors contain a high proportion (>90–95%) of O4+ cells (Supplementary Fig. 2—control condition panel) as we have previously shown for adult donor derived cells^57^. Previous flow cytometry studies indicated that the OLs express the late antigen MOG^28^. We did not detect PDGFRα+ cells in these cultures at the time of functional injury studies.

**Fig. 1:**
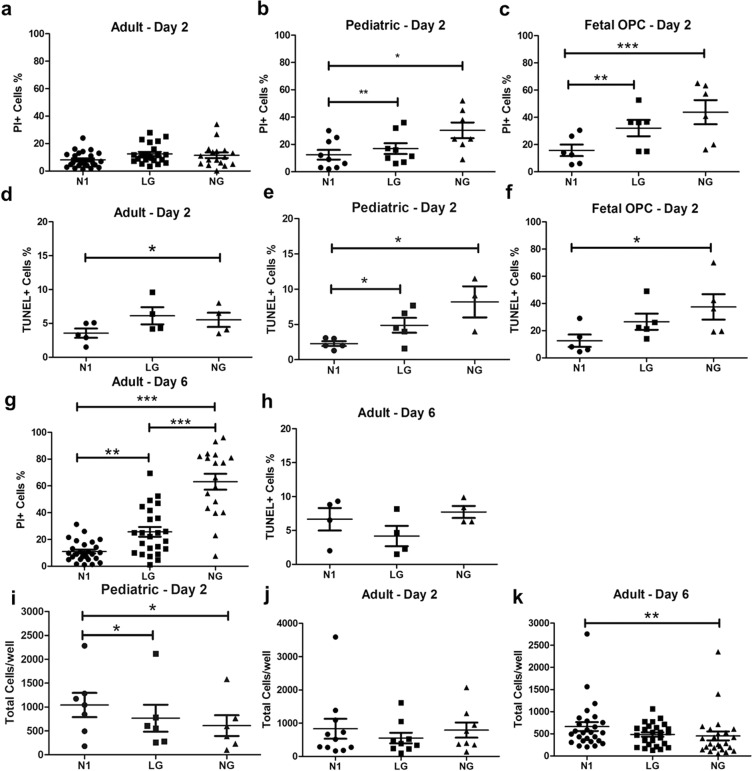
In vitro cell death response of adult, pediatric and fetal samples derived human OLs to glucose deprivation conditions. **a**–**c** % PI+ cells in 2-day cultures under N1, LG, and NG conditions. **a** Adult OLs show no significant increase in % PI+ cells under LG/NG conditions. **b** Pediatric OLs show significant increase in % PI+ cells under LG and more so under NG conditions. **c** Fetal O4+ cells show high levels of % PI+ cells under LG and more so under NG conditions. **d**–**f** % TUNEL+ cells in 2-day cultures under N1, LG, and NG conditions. **d** Adult OLs show no significant increase in %TUNEL+ cells under LG/NG conditions. **e** Pediatric OLs show significant increase in %TUNEL+ cells under NG condition but lower than % PI+ cells. **f** Fetal O4+ cells show high levels of %TUNEL+ cells comparable to % PI+ cells under LG and NG conditions. **g**–**h** % PI+ and TUNEL+ adult OLs maintained in culture for 6 days. There are increased levels of % PI+ cells under LG and NG conditions (**g**), while there is no significant increase in %TUNEL+ cells (**h**). **i**–**k** Cell number/well in 2-day cultures under N1, LG, and NG conditions. Pediatric OLs at 2 days under LG and NG conditions and adult OLs at day 6 under NG conditions show significant decrease in cell numbers/well compared to N1 conditions. For some samples only LG or NG experimental conditions could be evaluated; all were compared to N1 control conditions. Each point in the graph corresponds to an independent biological sample. Mean ± SEM for each condition shown in the figure. Statistical significance was verified by Student’s *t*-test: *(<0.05), **(<0.01), ***(<0.001).

As shown in Fig. 1a, for OLs derived from the human adult brain, there was no significant cell death (% propidium iodide (PI) positive cells) after 48 h of culture in LG or NG conditions. In contrast, at the same time point, significant cell death (% PI positive cells) was detected in OLs derived from pediatric donors under LG or NG conditions (Fig. 1b, illustrated in Supplementary Fig. 2—LG and NG conditions panels). The mean % PI+ cells was significantly higher in the pediatric OLs compared to adults in NG conditions (30.3 ± 5.7% vs 11.5 ± 2.1%; *p* < 0.05) (Fig. 1a, b). Total cell numbers were also significantly reduced in the pediatric sample (Fig. 1i). A sub-analysis based on donor age suggested increased susceptibility in the younger donors (<age 5 vs >age 5, 23 ± 5% vs 8 ± 1%; *p* < 0.05). The pediatric OLs were however more resistant to cell death (% PI+ cells) compared to O4+ cells isolated from fetal human brain samples under LG conditions at 2 days (Fig. 1c) (16.9 ± 4.0% vs 32.1 ± 6.0%; *p* = 0.06). For the pediatric samples, the proportion of TUNEL+ cells was also statistically higher in LG and NG conditions compared to N1 conditions (Fig. 1e); however, the percentage of TUNEL+ cells under NG conditions was significantly lower than the percentage of PI positive cells (8.2 ± 2.2% vs 30.3 ± 5.7%; *p* < 0.05) (Fig. 1b, e). For the fetal cells, % TUNEL+ cell was comparable to the % PI+ cells (Fig. 1c, f) (37.5 ± 9.3% vs 43.8 ± 8.9%). As shown in Fig. 1g, h, prolonged (6 day) culture of the adult OLs under LG and NG conditions resulted in a higher % of PI+ cells that was not associated with any significant increase in % TUNEL+ cells. Cell numbers were also reduced under NG vs N1 conditions at day 6 in the adult cell cultures (Fig. 1j, k).

### Identification of OL lineage subsets by whole-cell scRNA seq of immediately ex vivo cells

**Fig. 2:**
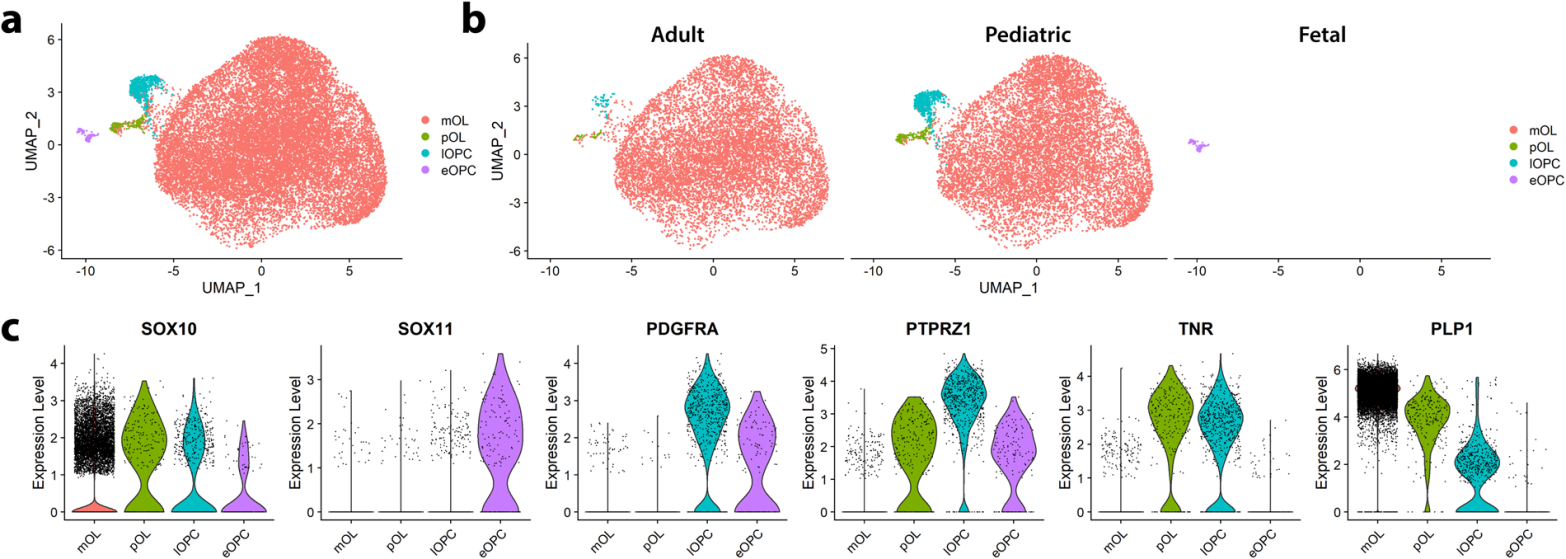
Sub-populations of human OL lineage cells as defined by whole scRNA sequencing. OL lineage cells derived from whole-cell sequencing of fetal, pediatric, and adult tissue samples cluster into distinct OL lineage subpopulations. **a** Uniform manifold approximation projection (UMAP) plots of pooled OL-lineage cells, visualizing 4 clusters: mOL, pOL, lOPC, and eOPC. Individual dots on UMAP plots represents single cells. **b** UMAP plots of OL-lineage cells split by age-group. **c** Violin plots showing relative expression level across clusters of selected marker genes, where expression levels are z-scored normalized average expression and gray dots indicate individual cells expressing the marker. Number of independent biological samples by age group: 4 adult, 7 pediatric and 4 fetal.

In order to identify sub-populations of OL lineage cells that may have distinct cell death responses, we applied unbiased cluster analysis to the pooled OL-lineage cells across a range of ages (Fig. 2a, b). Figure 2c presents violin plots showing relative expression levels across clusters of selected marker genes. In addition to the three subpopulations mature OLs (mOLs), late OPCs (lOPCs), and early OPCs (eOPCs) as identified in our previous report^33^, we identified an additional *PDGFRA- PTPRZ1*+ *MBP*+ population, here labeled as committed pre-OLs (pOLs). A population expressing both immune and oligodendrocyte markers, akin to that described by Jäkel et al.^58^, was also identified but not included in this analysis (Supplementary Fig. 3a).

As seen in Fig. 2a, b, mature OLs comprised the large majority of OL lineage cells in both adult and pediatric samples although progenitors were apparently more abundant in the pediatric samples (quantified in Supplementary Fig. 3b). eOPCs were derived entirely from fetal samples.

### Contributions of pro- and anti-apoptotic BCL-2 family members to OL susceptibility to metabolic injury

To identify molecular signatures that would help understand the basis for the observed age-related differences in resistance to metabolic insult, we first considered the baseline expression of genes encoding pro- and anti-apoptotic BCL-2 family members in the OLs in our adult and pediatric samples.

**Fig. 3:**
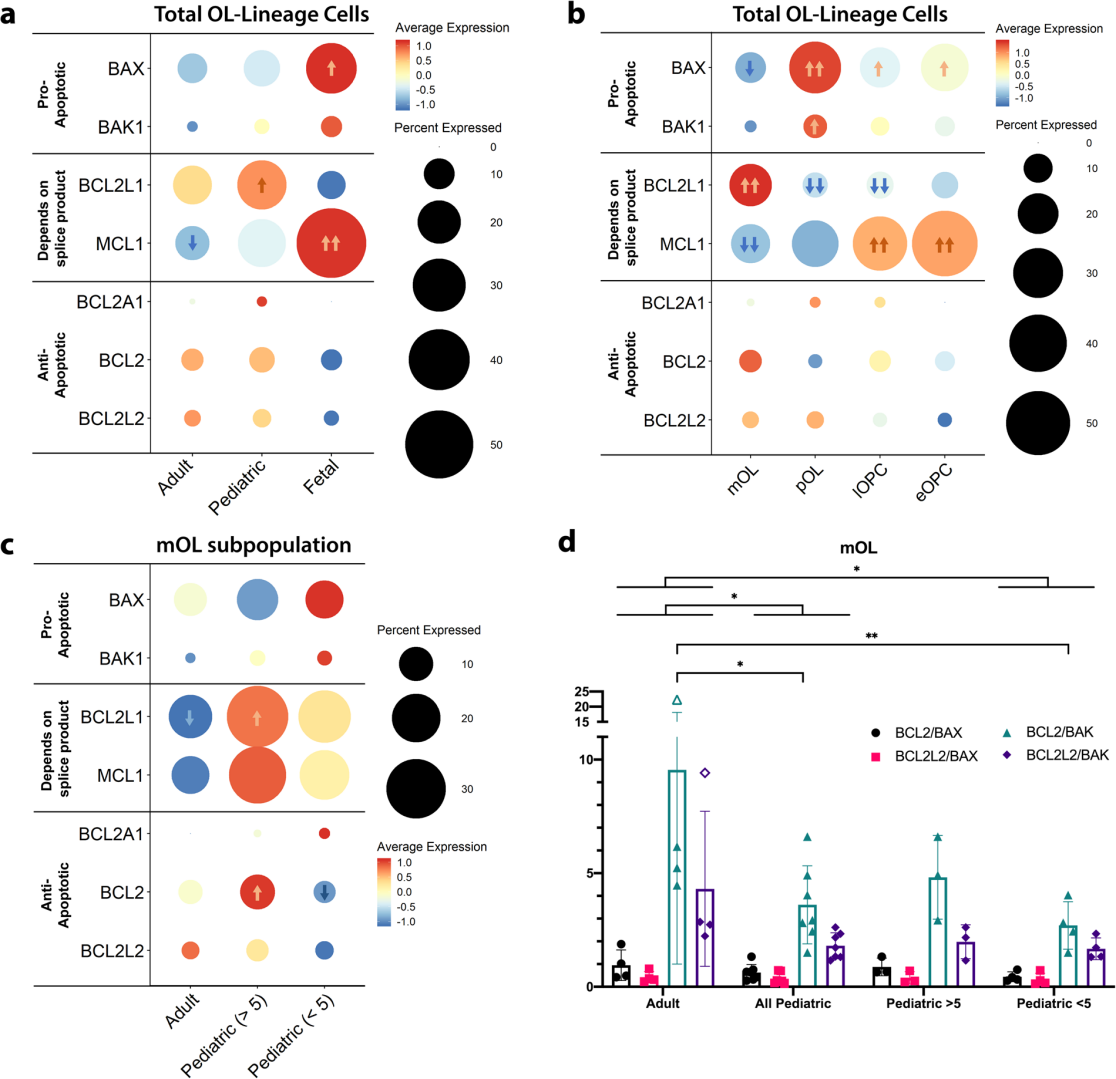
Differential baseline BCL-2 family gene expression in adult, pediatric and fetal OL-lineage cells. **a**–**c** Relative expression of pro and anti-apoptotic BCL-2 family genes across OL lineage spectrum in relation to age and lineage. Scale is z-scores of averaged normalized gene expression across the cell types or age groups; percent expressed indicates the percentage of OL-lineage cells in the category on the *x*-axis expressing the gene. Arrows indicate up- (orange) or down- (blue) regulation (adj. *p* < 0.05) against all other groups, where one arrow |logFC | > 0.1, two arrows |logFC | > 0.25, by Wilcoxon rank sum differential expression testing with Bonferroni correction. **d** Data indicates the ratios of average expression of individual pro- and anti-apoptotic genes for mature OLs. Data under all pediatric are combined from pediatric >5 and pediatric <5. Open circles indicate outliers by Grubbs’ test. Comparison of summated and individual ratios between adult and all pediatric donors was done by two-way ANOVA with Sidak’s correction for multiple comparisons both with and without the outliers, *p < 0.05. Age group comparisons of summated and individual ratios between adult, pediatric >5, and pediatric <5 was done by two-way ANOVA with Tukey’s correction for multiple comparisons, both with and without the outliers, *p < 0.05, **p < 0.01. Number of independent biological samples by age group: 4 adult, 7 pediatric (3 pediatric >5, 4 pediatric <5) and 4 fetal.

Our initial analysis across age groups includes pooled total OL-lineage cells from all our donor samples as for some subsets there were insufficient numbers of cells to compare between individual samples. We divided the BCL-2 family genes into pro-apoptotic (*BAX, BAK1*) and anti-apoptotic (*BCL2A1, BCL2, BCL2L2*) categories, and further separated genes whose product activity depends on alternatively spliced products (*BCL2L1, MCL1*)^59,60^ (Fig. 3a–c). *BCL2L10* is not included as it was not captured due to dropout events in sequencing. Actual normalized expression values are presented in Supplementary Dataset 1. As presented in Fig. 3a, for the total OL cluster, the fetal derived OL cells expressed higher levels of pro-apoptotic gene *BAX* as compared to pediatric and adult donor cells. Comparison of OL sub-populations defined by differentiation stage indicates skewing to greater anti- vs pro-apoptotic gene expression with cell differentiation (Fig. 3b), particularly on comparison of mOLs and pOLs with lOPCs and eOPCs. As shown in Supplementary Fig. 4a, comparisons of different lineages within the pediatric age group indicate that there is skewing to greater anti- vs pro-apoptotic gene expression with cell differentiation. We are unable to draw a statistical conclusion from the adult cohort as the numbers of progenitor cells are too limited (Supplementary Fig. 4b).

Figure 3c presents the expression patterns for mature OLs from pediatric and adult samples, sub-dividing the pediatric group into <5 and >5-year subgroups, coinciding with our functional in vitro studies. This analysis suggested an increase in anti- vs pro-apoptotic BCL-2 family gene expression pattern with age. Figure 3d presents ratios of expression of individual pro- and anti-apoptotic genes calculated from individual donors, as well as the pooled ratio of these genes. The pooled data indicates a significant increase in the anti- vs pro-apoptotic gene expression ratio in the adult compared to the overall pediatric samples. The main difference from the adult group is derived from the younger cohort.

We could not identify age and differentiation stage-related expression patterns in BCL-2 family genes whose product activity depends on alternatively spliced products. Additional data suggest that age-related differences in anti- vs pro-apoptotic BCL-2 family gene expression is also noted in lOPCs and pOLs though these did not reach significance (Supplementary Fig. 4c, d).

**Fig. 4:**
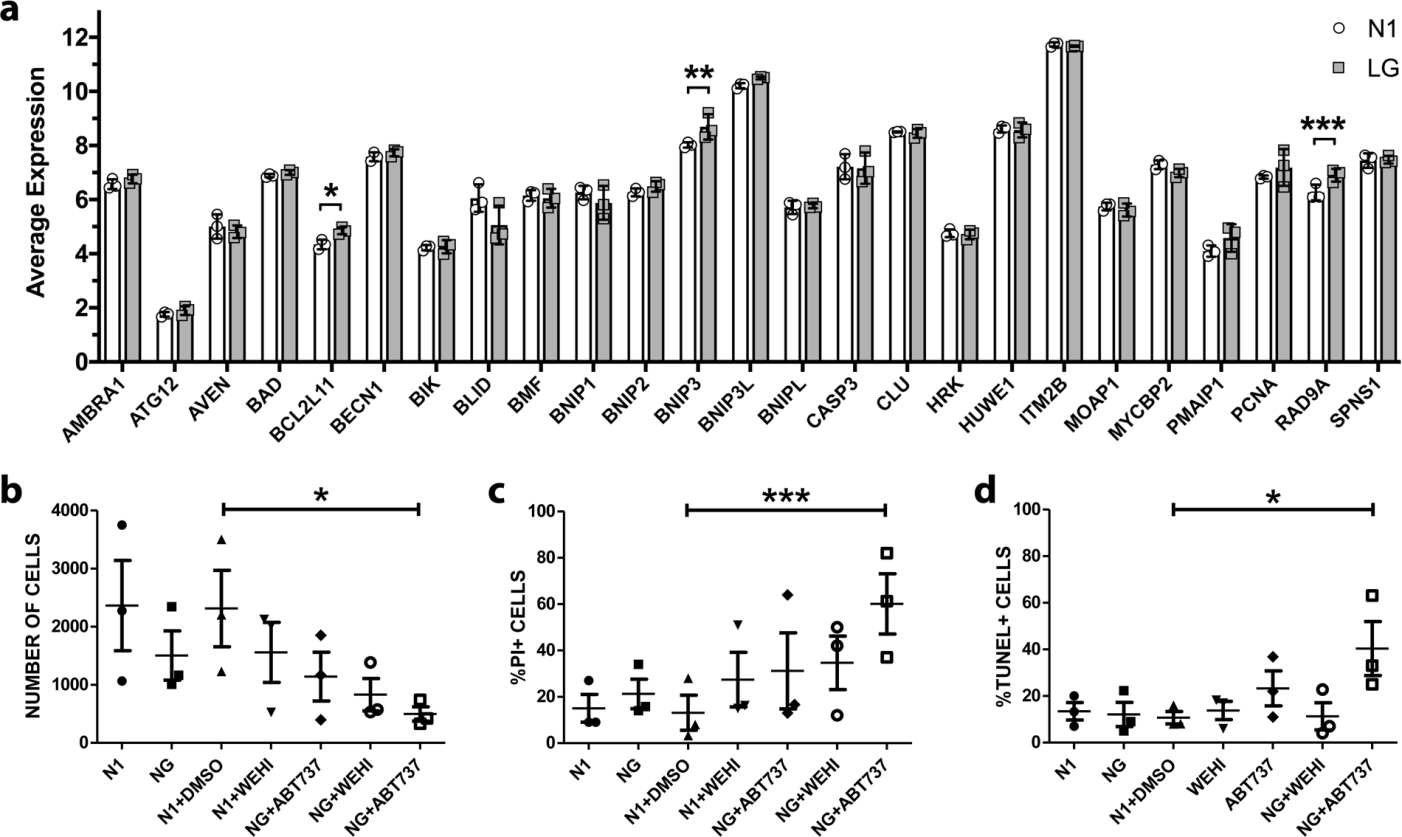
Contribution of BCL-2 family genes to protecting human adult OLs from metabolic injury. **a** Changes in RNA expression of the BH3-only genes in human adult OLs due to glucose deprivation. Average RNA expression levels of members of the BH3-only family in media containing low concentration of glucose (LG) or in optimal culture media (N1) derived by microarray analysis of human adult OLs. *N* = 3 independent biological samples. **b**–**d** Effects of inhibition of the BCL-2 family on cell death of human adult OLs. Cells were cultured in optimal culture media (N1) and in media with no glucose (NG) and treated with the specific inhibitor of BCL-XL WEHI-539 and the non-specific inhibitor ABT737 in N1 and NG media for two days. Cells treated only with DMSO were used as the vehicle control. *N* = 3 independent biological samples. Data are presented as (**b**) number of cells present; **c** %PI+ cells and **d** %TUNEL+ cells. Mean ± SEM for each condition in the figure. Significant levels for the ANOVA/Dunnett’s test for paired samples with DMSO as reference: *(<0.05), **(<0.01).

To address the hypothesis that the observed lack of apoptotic cell death of the adult donor cells may reflect the activation state of BH3-only molecules, we re-examined our previously described microarray of adult human OLs under LG conditions^19^ for the expression of 25 previously characterized BH3-only subfamily members^61^. As shown in Fig. 4a, there are significant increases in the expression of *BCL2L11, BNIP3*, and *RAD9A* under LG condition for 48 h; at this time point, there was no significant change in expression of anti-apoptotic and pro-apoptotic genes (Supplementary Fig. 5) and no significant cell death (Fig. 1). These results suggest that the intrinsic apoptotic pathway was initiated by activation of some BH3-only molecules, but then inhibited by anti-apoptotic proteins.

To directly investigate the potential contribution of BCL-2 family anti-apoptotic molecules to cell survival in human adult OLs, we used ABT-737, an inhibitor of multiple BCL-2 family anti-apoptotic molecules (mainly BCL-XL, BCL-2, and BCL-W) and WEHI-539, a specific inhibitor of BCL-XL^62,63^. Addition of ABT-737 to adult donor OLs significantly increased cell death in NG compared to control conditions, as measured by reduced number of surviving cells and increased % PI+ cells (Fig. 4b, c). Cell process extension was also reduced (Supplementary Fig. 6). There was also a significant increase in % TUNEL+ cells (Fig. 4d) indicating that the adult OLs have the capacity to activate the BCL-pro-apoptotic cascade if anti-apoptotic proteins are inhibited. The relative lack of effect of WEHI-539 suggests that inhibiting BCL-XL alone is not sufficient to inhibit apoptosis.

### Contribution of autophagy pathway genes to age-related OL susceptibility to metabolic injury

**Fig. 5:**
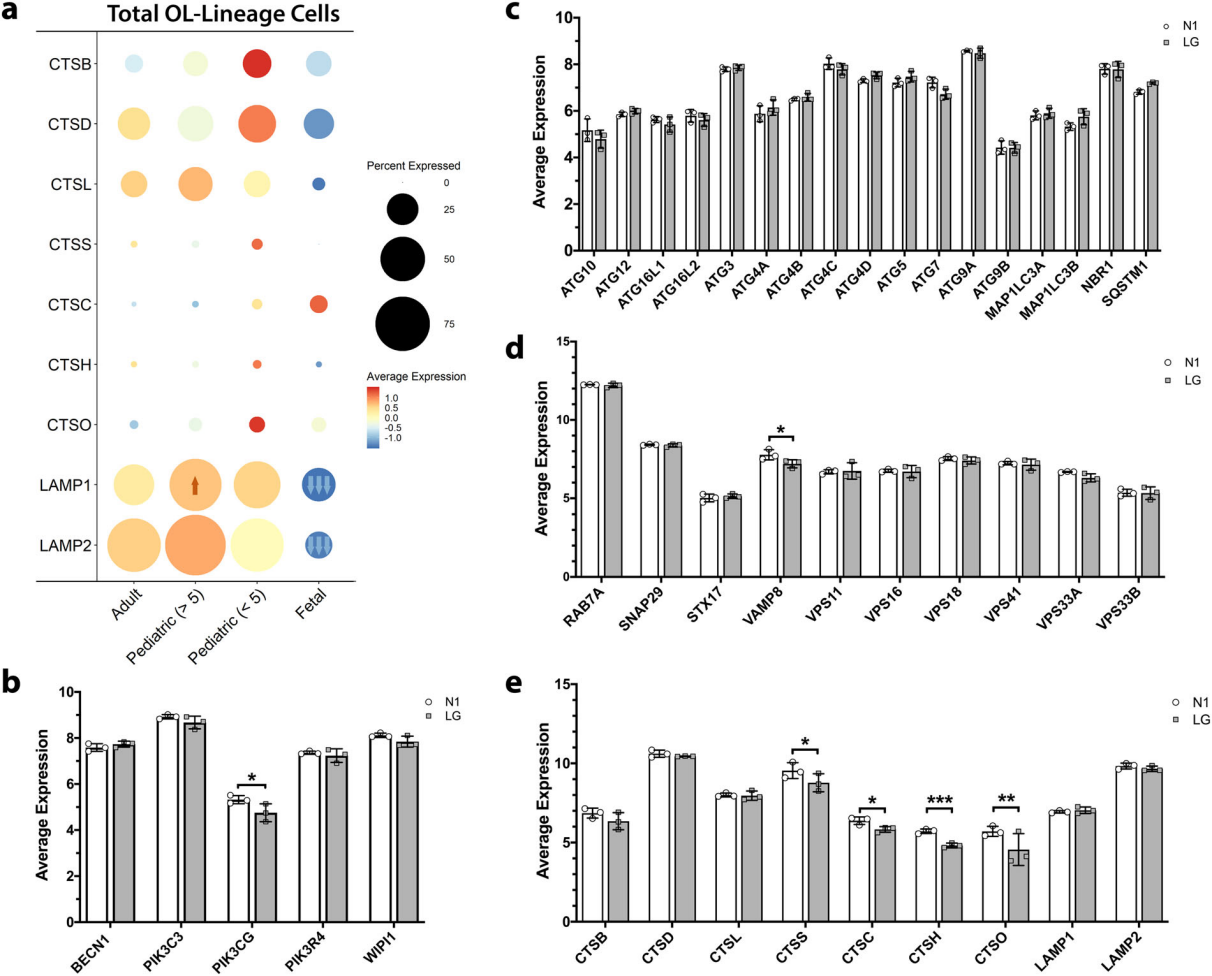
Differential baseline expression of genes of the autophagy pathway in adult, pediatric, and fetal OL-lineage cells. **a** Relative expression of lysosome formation genes of the autophagy pathway in total OL lineage cells from adult, pediatric, and fetal samples. Scale is z-scores of averaged normalized gene expression across the age groups; percent expressed indicates the percentage of OL lineage cells in the age group expressing the gene. Arrows indicate up- (orange) or down- (blue) regulation (adj. *p* < 0.05) against all other groups where one arrow is |logFC | > 0.1, two arrows |logFC | > 0.25, three arrows |logFC | > 0.99, by Wilcoxon rank sum differential expression testing with Bonferroni correction. Number of independent biological samples by age group: 4 adult, 7 pediatric and 4 fetal. **b**–**e** Relative expression of genes involved in nucleation of autophagosomes (**b**), elongation of autophagosomes (**c**), fusion of autophagosomes and lysosomes (**d**) and lysosome formation (**e**) in optimal conditions (N1) and low glucose (LG) in adult OLs. mRNA expression was measured by microarray analysis with *N* = 3 independent biological samples from adult donors. Mean ± SEM for each condition in the figure. Statistical significance was verified by Student’s *t*-test: *(<0.05), **(<0.01), ***(<0.001). *SQSTM1* encodes for p62 and *MAP1LC3B* encodes for LC3-II.

To determine whether differences in the relative baseline expression of autophagy genes contributed to the observed age and differentiation linked injury responses, we examined the expression of a select number of genes contributing to the autophagy response in relation to these variables. As reviewed in^49,52^ autophagy can be divided into four stages: initiation, nucleation, elongation and fusion. As shown in Fig. 5a, we detected a trend to decreased expression of lysosome-related genes in fetal total OL-lineage cells as compared to pediatric and adult OLs; in particular, *LAMP1* and *LAMP2* were significantly downregulated. Consistent age-related differences in expression of genes involved in autophagy initiation, nucleation, and elongation were not detected (Supplementary Fig. 7).

We did not detect differences in expression of autophagy genes involved in autophagy initiation in OLs under 48 h of glucose deprivation with the exception of *PIK3CG* that is downregulated (Fig. 5b). We did not detect a significant change in the expression levels of the elongation genes with glucose deprivation alone (Fig. 5c). Expression of genes encoding LC3 (*MAP1LC3B*) and p62 (*SQSTM1*) that were measured in the protein assays were suggestively increased (Fig. 5c). Regarding the expression of genes involved in fusion of lysosomes and autophagosomes (Fig. 5d), only *VAMP8* is downregulated under low glucose. Among the genes that are involved in the lysosome formation (Fig. 5e), cathepsins C, H and O are downregulated although there is no change in cathepsins B, D, and L.

**Fig. 6:**
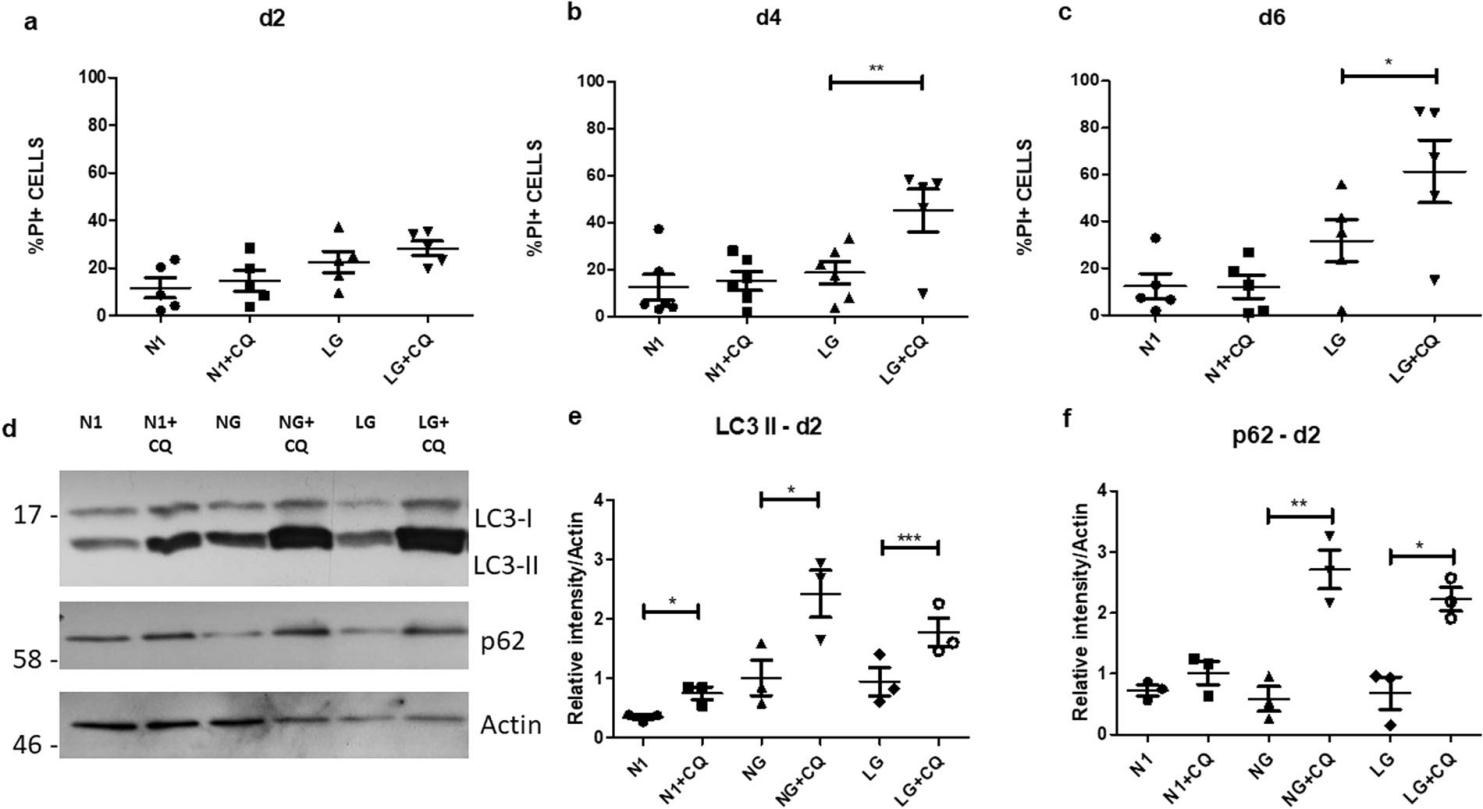
Contribution of the autophagy pathway to protecting OLs from metabolic injury. **a**–**c** Protective effects of autophagy in human adult OLs. Cell death measured by % PI+ cells of human adult OLs cultured in optimal culture media (N1), in low glucose media (LG) and in combined treatment with chloroquine (N1 + CQ; LG + CQ) after two (**a**), four (**b**) and six days (**c**). Addition of CQ results in increase % PI+ cells at day 4 and 6. *N* = 5 independent biological samples. **d**–**f** Representative western blot for the combined treatment of N1, LG, and NG with and without chloroquine after two days (*N* = 3 independent biological samples) (**d**) and quantification of the relative expression compared to actin of LC3 II (**e**) and p62 (**f**). Mean ± SEM for each condition in the figure. Statistical significance was verified by ANOVA/Tukey test: *(<0.05), **(<0.01), ***(<0.001).

To determine whether glucose deprivation conditions at a time prior to cell death were inducing a potential protective autophagy response, we treated cultures with chloroquine, which inhibits the fusion of autophagosomes with lysosomes^64^ (Fig. 6a–c). We observed that chloroquine did not increase cell death in the human OLs under optimal culture conditions (N1) but significantly increased cell death (% PI+ cells) in glucose deprivation conditions (Fig. 6a–c). Cell process extension was also reduced (Supplementary Fig. 6). To further investigate the engagement of autophagy in the response of the OLs to LG/NG conditions and the effect of chloroquine, we analyzed the protein levels of the autophagosome components LC3 and p62 by western blot. We detected an increase in the levels of these markers in these conditions when combined with chloroquine (Fig. 6d–f). This was not seen when cells were cultured in optimal conditions. These results indicate that autophagy is activated in glucose deprivation conditions. Accumulation of autophagosomes in these cells indicate an impairment in the fusion with lysosomes induced by chloroquine. These combined functional and Western blot studies support the early protective effects of the autophagy response.

## Discussion

Our data identify BCL-2 family and autophagy pathways as contributors to age-linked responses of human OL lineage cells to metabolic injury. The viability of the cells isolated immediately from the surgical samples allowed us to perform both whole-cell scRNA seq, which includes cytoplasmic RNA in contrast to single nucleus studies, and complementary in vitro functional assays. Samples were obtained from normal appearing tissue, within the corridor required to access the pathologic site. The status of the samples was confirmed by histopathologic analysis. The metabolic insult used was designed to model focal ischemic conditions found in MS lesions and in the penumbra of focal pediatric ischemic/hemorrhagic events.

The functional in vitro injury data indicate that under the stress conditions used (combining LG/NG with reduced nutrient supplements), human fetal OL lineage cells (O4+) were highly susceptible to cell death (within 24–48 h), with the majority of cells being TUNEL+. The OLs derived from the pediatric age samples were more resistant than the fetal cells but more susceptible to injury than the adult cells. The pediatric OLs showed measurable, although limited, apoptosis. Our data that active cell death pathways are prominent in early human OPCs in response to metabolic insults are supportive of findings from rat models of hypoxic cerebral hypoxia-ischemia. The latter showed that apoptotic mechanisms were several-fold more pronounced in immature than in juvenile and adult brains^29,65,66^. For adult OLs, consistent with our previous report^8^ the delayed cell death was not linked to induction of active cell death pathways.

The bioinformatics data provides insight into the molecular basis for the age-related functional injury responses that we detected. We selected representative members of the BCL-2 pathway that are well documented to be pro-or anti-apoptotic. Using the total OL population, we found that the highly injury susceptible fetal cells, containing the early OPC population, expressed the highest levels of pro-apoptotic genes. With OL differentiation, there is skewing to greater anti- vs pro-apoptotic gene expression. Our observation that pediatric donor mature OLs cells still exhibit a relative increase in the expression of pro- vs anti-apoptotic genes compared to the adult derived cells, is consistent with our observed age-related susceptibility to injury. Our in vitro studies showing that blocking anti-apoptotic genes results in significant apoptotic cell death under the LG conditions, indicate the functional relevance of the relative skewing to anti- vs pro-apoptotic gene expression in OLs. Our data suggest that anti-apoptotic genes are impeding the BH3-only family molecules, observed to be activated, from inducing the apoptosis pathway. BH3-only family members can be triggered by multiple pathologic conditions, including hypoxia^38,39,67^.

The cell death induced by the metabolic insult used in this study is likely closely linked to energy failure. We previously documented immunocytochemically that the metabolic conditions used in the current study increased LC3 expression, an indicator of autophagy activation in adult human OLs^8^. We now confirm by Western blotting for p62 and LC3-II that autophagy is activated in these cells and demonstrate that the response is initially protective, based on the increased cell death in the presence of chloroquine. The bioinformatics data derived from immediately ex vivo cells indicate that the fetal OL lineage cells that are highly susceptible to metabolic stress, have reduced expression of the lysosome forming genes, which are an integral part of the autophagy pathway. Our observations seem compatible with those of Neumann et al. that autophagy pathway gene expression is upregulated in A2B5 antibody selected progenitor cells in aged rats; these cells overall had reduced stem cell properties^55,56^.

The microarray data from the adult human OLs cultured in LG for 2 days (the time when autophagy is activated) indicate that while expression of most genes involved in encoding the autophagy pathway are unchanged, a limited number of genes were downregulated. These included *PIK3CG*, whose gene product inhibits autophagy initiation^47^, and *VAMP8*, which encodes a SNARE protein that is necessary for the attachment of lysosomes to autophagosomes^52^. Among the genes involved in lysosome formation, cathepsins C, H, and O are downregulated although there is no change in cathepsins B, D, and L. This downregulation may reduce the digestion capacity of lysosomes, although it is possible that the overall concentration of cathepsins would be sufficient. p62 and LC3 are suggestively upregulated. We did not detect a significant change in the expression levels of elongation genes, including those encoding p62 and LC3. We consider that some downregulation of genes under our LG conditions can reflect overall reduction in metabolic activity of the cells as we have previously shown using the Seahorse bioanalyzer^7^. Our overall results suggest that the observed autophagy activation most reflects post-translational changes.

Overall, our study provides molecular and functional data to document the susceptibility of human OL lineage cells to metabolic injury and elucidates the role of age and OL lineage cell maturity level in this process. Defining the mechanism of cell death can direct therapeutics aimed at protecting cells that may initially survive acute or chronic insults across the age spectrum, enhancing the possibility for their participation in subsequent myelin repair^9,10^.

## Methods

### Human cell samples

Brain tissue samples were obtained from surgical procedures. Anonymized adult samples were obtained via the Department of Neuropathology at the Montreal Neurological Institute and Hospital (MNI) and pediatric samples from the Montreal Children’s Hospital with written consent from families. 2nd trimester (14–17 weeks) fetal samples were obtained from the University of Washington Birth Defects Research Laboratory (MP-37-2014-540; 13-244-PED; eReviews_3345). Demographics of the pediatric patients are given in Supplementary Table 1. Adult donors ranged in age from 30–65 years; all had non-tumor related focal epilepsy. Use of pediatric and adult tissues where approved by the MNI Neurosciences Research Ethics Board (Protocol ANTJ 1988/3) and the use of pediatric tissues approved by the Montreal Children’s Hospital Research Ethics Board.

### Cell isolation

Tissues from the surgical corridor from the adult and pediatric samples were collected into CUSA bags. Tissue derived from the CUSA bags was subjected to trypsin digestion followed by Percoll gradient centrifugation in order to obtain a myelin-depleted whole-cell fraction comprised mainly of OLs and microglia with few if any astrocytes or neurons. The scRNA seq data from the adult and pediatric surgical samples were derived from sequencing this total cell population. For in vitro studies of the adult and pediatric samples, we derived an enriched OL population by adhering the total cell population overnight to remove adherent microglia. Fetal cell isolation does not require a Percoll gradient step but in order to enrich for OL cells for our functional assays, we used immuno-magnetic beads coated with O4 antibody (IgM)^28^ to collect rare (1–5%) O4+ cells.

### Cell culture

After selection, cells were plated in 96-wells or 24-wells plates coated with poly-lysine and extra-cellular matrix at a density of 3 × 10^4^ cells (96-wells plate) or 1 × 10^6^ cells (24-wells plate) per well. Cells were cultured in DMEM-F12 media supplemented with N1 (Sigma, Oakville, ON, Canada. For metabolic deprivation experiments, cells were cultured in DMEM containing 0.25 g/l of glucose (LG) or with no glucose added (NG).

### Immunocytochemistry

Cells were live stained with propidium iodide (PI; Invitrogen) (1:200) for cell viability measurement and with O4 monoclonal antibody (R&D Systems, Minneapolis, MN) (1:200) for 15 min at 37 °C and then fixed with 4% paraformaldehyde for 10 min in room temperature. Goat anti-mouse IgM Cy3 (1:500) was used as secondary antibody to O4, 30 min at room temperature. Cells were stained with a commercial TUNEL kit (Promega, Madison, WI). Cell nuclei were stained with Hoechst 33258 (1:1000). Inhibitors used in these experiments were: Chloroquine (10 µM; Sigma, Oakville, ON, Canada), WEHI-539 (10 µM; Selleckchem, Houston, TX) and ABT-737 (10 µM; Selleckchem, Houston, TX).

### Western blot

SDS-PAGE was run with 5–10 µg of protein in each sample in 15% acrylamide gels. Proteins were transferred to a PVDF membrane. The membranes were blocked with 5% milk and probed with the primary antibodies overnight at 4 °C. Bands were visualized with horseradish peroxidase-conjugated secondary antibody used in conjunction with an ECL Western blot detection kit (Cell Signaling, Danvers, MA). Primary antibodies used were LC3B (#2775 Cell Signaling, Danvers, MA), SQSTM1/p62 (#5114 Cell Signaling, Danvers, MA) and β-actin (Sigma, Oakville, ON, Canada); the dilution used in all primary and secondary antibodies was 1:1000.

### Single-cell RNA sequencing

Fetal, pediatric, and adult tissue samples were sent for sequencing at the McGill University and Génome Québec Centre. RNA sequencing libraries were prepared with 10X Chromium v2.0 and single cell RNA sequencing (scRNAseq) was performed on the Illumina HiSeq4000 PE75 sequencer. A droplet-based sequencing method by 10X Chromium was used to obtain single-cells. The 10XGenomics CellRanger pipeline was used to demultiplex cell and unique molecular identifier barcodes, and to align reads to the GRCh38 human reference genome. All subsequent analyses were primarily done using the Seurat (v3.1) R package^68–71^.

The standard Seurat pipeline for quality control, gene expression normalization, batch-effect correction, clustering, and differential expression analysis was used. For each sample, dead cells were filtered by removing cells with >5% mitochondrial gene content. Low quality cells and cell multiplets were removed by excluding cells with unique feature counts of less than 200 or over 2500 respectively. Expression counts were then natural log-normalized and scaled. A subset of the top 2000 highly variable features for each sample were identified to be prioritized during integration for batch correction, to adjust for sample-driven clustering. Integration was done using a method described by Stuart et al.^69^. Briefly, cell pairwise correspondences across datasets used to “anchor” the datasets were identified after canonical correlation analysis (CCA) reduction and L2-normalization to canonical correlation vectors. Then, “correction” vectors for each cell were calculated and expression values were transformed to integrate datasets together for the purposes of clustering. All samples of pediatric and adult donors were integrated together into one dataset; all samples of fetal donors were integrated into a second dataset. Up to extracting OL-lineage cells, fetal samples were processed separately as their cell-type makeup was dramatically different from that of the pediatric and adult samples due to the earlier described differences in tissue processing. Running integration and batch correction in samples where cell types are extremely imbalanced can lead to incorrect alignment^71^.

Principal component analysis (PCA) was used on each dataset to reduce the high-dimensional dataset to lower dimensions. For this analysis, genes highly associated with tissue dissociation were regressed to reduce the generation of artifacts in downstream analyses, such as creating a subpopulation that does not exist in vivo^70^. A shared-nearest neighbor graph was constructed based on the PCA analysis of each large dataset and the Louvain clustering algorithm was used several times to identify clusters at multiple different resolutions. To visualize the clusters in two-dimensional space, uniform manifold approximation and projection (UMAP) was used for non-linear dimensional reduction. The clustree R package was used to construct a clustering tree^72^. The clustering tree visualizes the movement of cells between clustering branches and was used to determine the optimal resolution for the stable clustering. A high degree of movement between branches indicates over-clustering. A clustering resolution of 1.5 was chosen for the integrated fetal dataset and 0.5 was chosen for the integrated pediatric/adult dataset.

Clusters in each dataset were annotated as OL-lineage cells using relative expression of canonical markers such as SOX10 for OL-lineage cells*; PDGFRA, PTPRZ1* for oligodendrocyte progenitor cells (OPCs); and *MBP, PLP1*, for mature OLs (mOLs). OL-lineage cells were extracted from each of the two integrated datasets and merged to create a final total OL-lineage dataset. Louvain clustering with clustering tree analysis was again applied on these total OL-lineage cells. A base resolution of 0.08 was chosen and one modification was made to this clustering solution. A higher resolution of 0.35 was used to separate a specific subpopulation as differing expression of canonical OL-lineage markers *PDGFRA, PTPRZ1, MBP* was apparent and those particular clusters remained very stable even at higher resolutions.

### Statistics and reproducibility

#### In vitro studies

All statistics were measured by the mean and the standard error of the mean. The quantity of independent biological samples, statistical tests used and level of significance are indicated in the figure legends.

#### Single cell RNA seq differential expression analysis

Differential expression (DE) analysis based on the Wilcoxon rank sum test was done on the uncorrected expression values. Results were adjusted for familywise error rate (FWER) with Bonferroni correction. Genes were considered to be significantly up- or downregulated if they had an average log fold change (logFC) ≥ 0.25, adjusted *p* value < 0.05; lowly up- or downregulated if they had an average logFC ≥ 0.1, adjusted *p* value < 0.05. For comparisons between age groups, genes that were differentially expressed between samples of the same age group were removed from the DE list of that age group to account for human biological variability between samples.

## Supplementary information

Supplementary Information

Description of Additional Supplementary Files

Supplementary Data 1

## Data Availability

Source data for figures are provided at Supplementary Data 1. Uncropped scans of western blot are shown in Supplementary Figs. 8–10. Microarray and RNA-seq data were deposited at GEO under accession number GSE160813. Immunocytochemistry images will be provided upon request.
