# Supplementary material for: Age-related injury responses of human oligodendrocytes to metabolic insults: link to BCL-2 and autophagy pathways
Source: Commun Biol. 2021 Jan 4;4:20. doi: 10.1038/s42003-020-01557-1 (PMC7782481; doi:10.1038/s42003-020-01557-1)
Supplement: Supplementary file 1 — Supplementary Information [file 42003_2020_1557_MOESM1_ESM.pdf]

Age-Related Injury Responses of Human Oligodendrocytes to Metabolic Insults: Link to BCL-2 and Autophagy Pathways

Milton Guilherme Forestieri Fernandes, Julia Xiao Xuan Luo, Qiao-Ling Cui, Kelly Perlman, Florian Pernin, Moein Yaqubi, Jeff Hall, Roy Dudley, Myriam Srour, Charles Couturier, Kevin Petrecca, Catherine Larochelle, Luke M. Healy, Jo Anne Stratton, Timothy E. Kennedy, Jack P. Antel

Additional Information

Following Supplemental Materials are available in this file

Supplementary Figures 1-10 (available in this file)

Supplementary Table 1 (available in this file)

Supplementary Dataset 1 (available in an separated excel file)

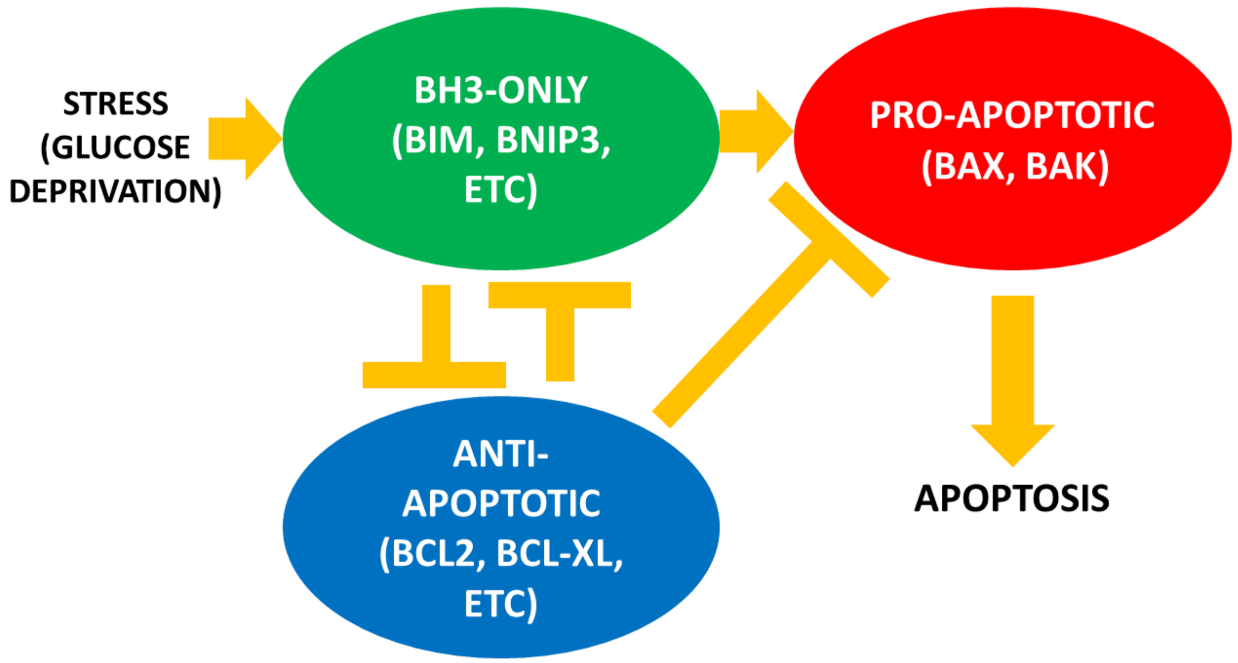

Supplementary Figure 1 – Role of BCL-2 sub-families interaction in the intrinsic apoptotic pathway. Illustration of how stress caused by glucose deprivation can activate the molecules of the BH3-only subfamily that in turn can directly activate the pro-apoptotic molecules or indirectly trigger apoptosis by inhibiting the anti-apoptotic subfamily. The anti-apoptotic molecules directly inhibit apoptosis by blocking the pro-apoptotic molecules or indirectly by blocking the action of BH3-only molecules on the pro-apoptotic molecules.

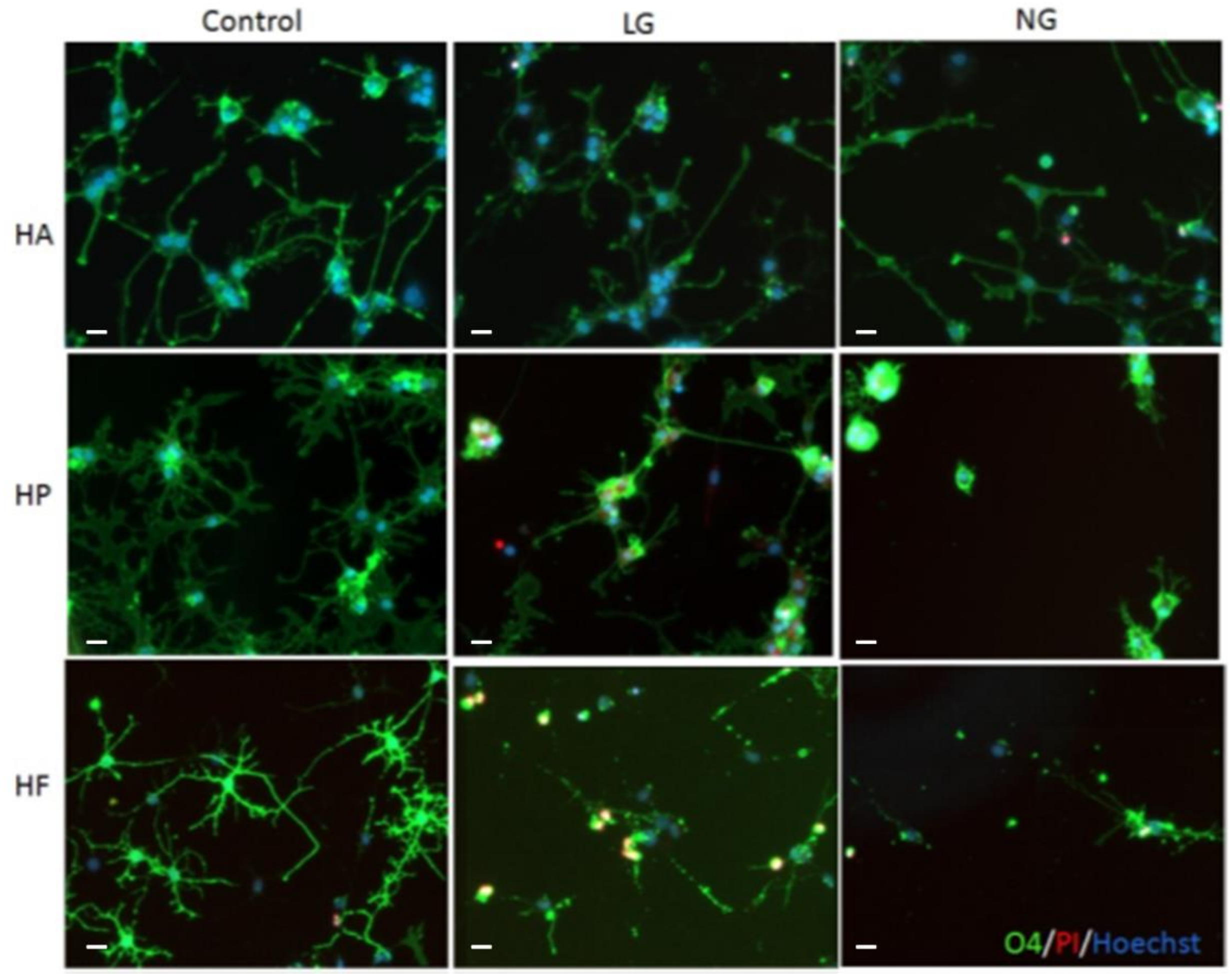

Supplementary Figure 2 - Expression of the oligodendrocyte marker O4 in adult, pediatric and fetal samples- dissociated culture of OLs derived from each of an adult (HA), pediatric (HP) and fetal (HF) sample under N1 control, LG and NG conditions for 2 days stained with anti-O4 antibody (green), propidium iodide (PI, red), and nuclear stain (Hoechst) blue, indicating presence of PI+ cells in pediatric and fetal O4+ cells in LG condition, and reduced cell numbers in NG condition. . Scale bar = 20 μm is shown in the bottom right, all other figures are in the same scale.

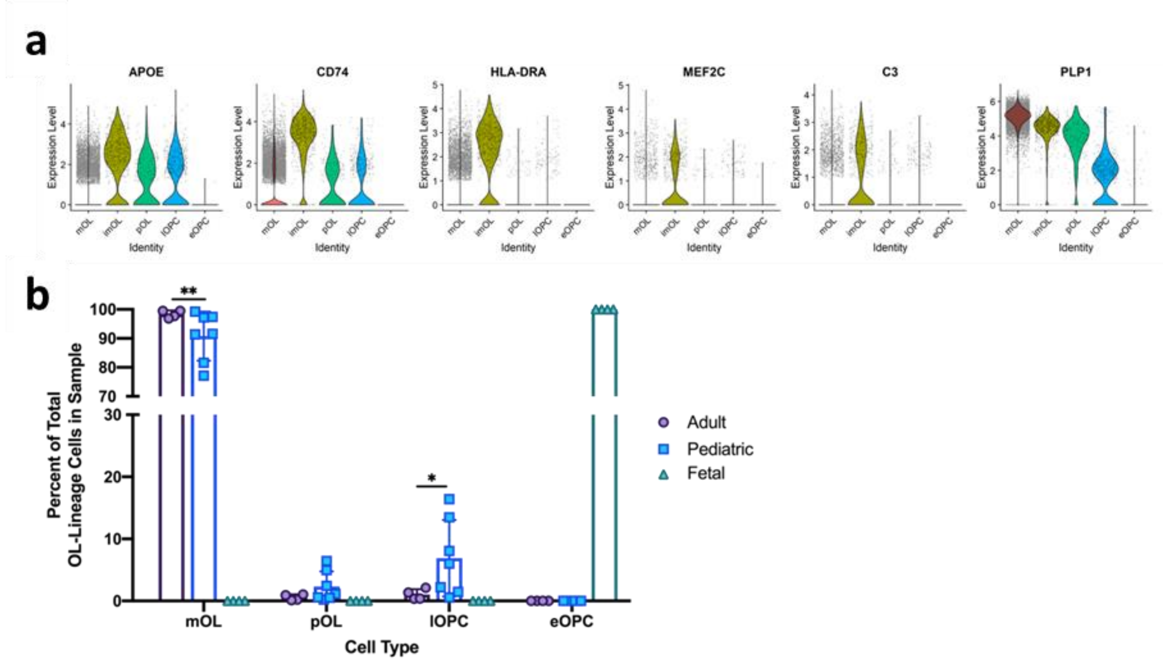

Supplementary Figure 3 – Expression of oligodendrocyte markers in the identified cells clusters. **a.** Expression of markers distinguishing immune-oligodendrocyte population identified by Jäkel et al. (2019). Expression levels are z-scored normalized average expression and grey dots indicate individual cells expressing the marker. **b.** OL lineage cell-type make-up differs in across age groups. Shown are percentage of cells in specific subpopulations out of total OL lineage cells for each sample by age-group. Two-way ANOVA with Tukey's correction for multiple comparisons was done, significance is adjusted $p<0.05$*, $p<0.01$**. Number of independent biological samples by age group: 4 adult, 7 pediatric and 4 fetal.

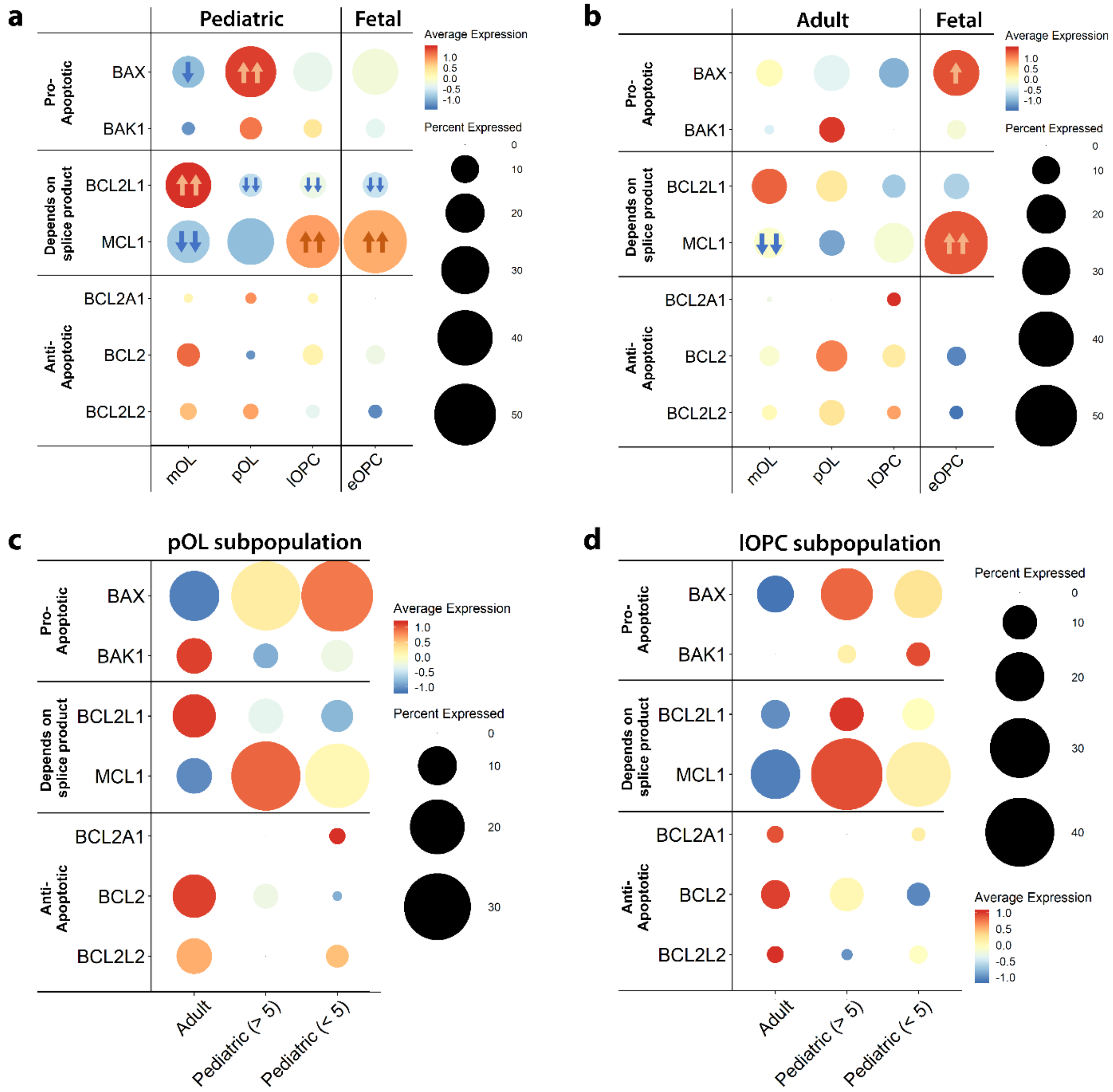

Supplementary Figure 4 – Relative expression of pro- and anti-apoptotic BCL-2 genes within age groups and OL-lineage cell types. Average expression in (a) OL-lineage cells within pediatric and fetal samples, (b) OL-lineage cells within adult and fetal samples, (c) pOLs across age groups, and (d) lOPCs across age groups. Scale is z-scores of averaged normalized gene expression across the cell types or age groups; percent expressed indicates the percentage of OL-lineage cells in the cell type or age group expressing the gene. Arrows indicate up- (orange) or down- (blue) regulation (adj. $p<0.05$) against all other groups on the x-axis, where one arrow $|logFC| > 0.1$, two arrows $|logFC| > 0.25$, by Wilcoxon rank sum differential expression testing with Bonferroni correction. Number of independent biological samples by age group: 4 adult, 7 pediatric (4 pediatric < 5; 3 pediatric >5), and 4 fetal.

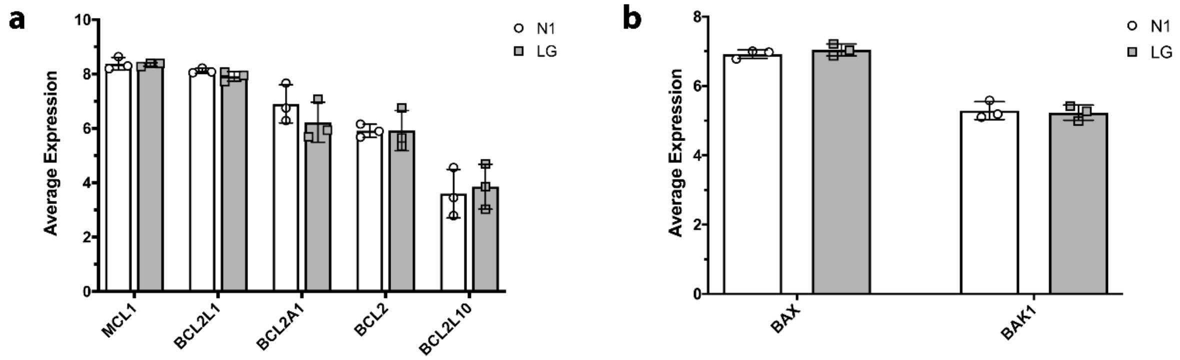

Supplementary Figure 5 - Changes of expression in the anti and pro-apoptotic molecules in human adult oligodendrocytes due to glucose deprivation. Average RNA level of the (**a**) anti-apoptotic and (**b**) pro-apoptotic molecules of the BCL-2 family in media containing low concentration of glucose (LG) or in optimal culture media (N1) derived by microarray analysis of adult human oligodendrocytes. N=3 independent biological samples. Mean±SEM for each condition in the figure.

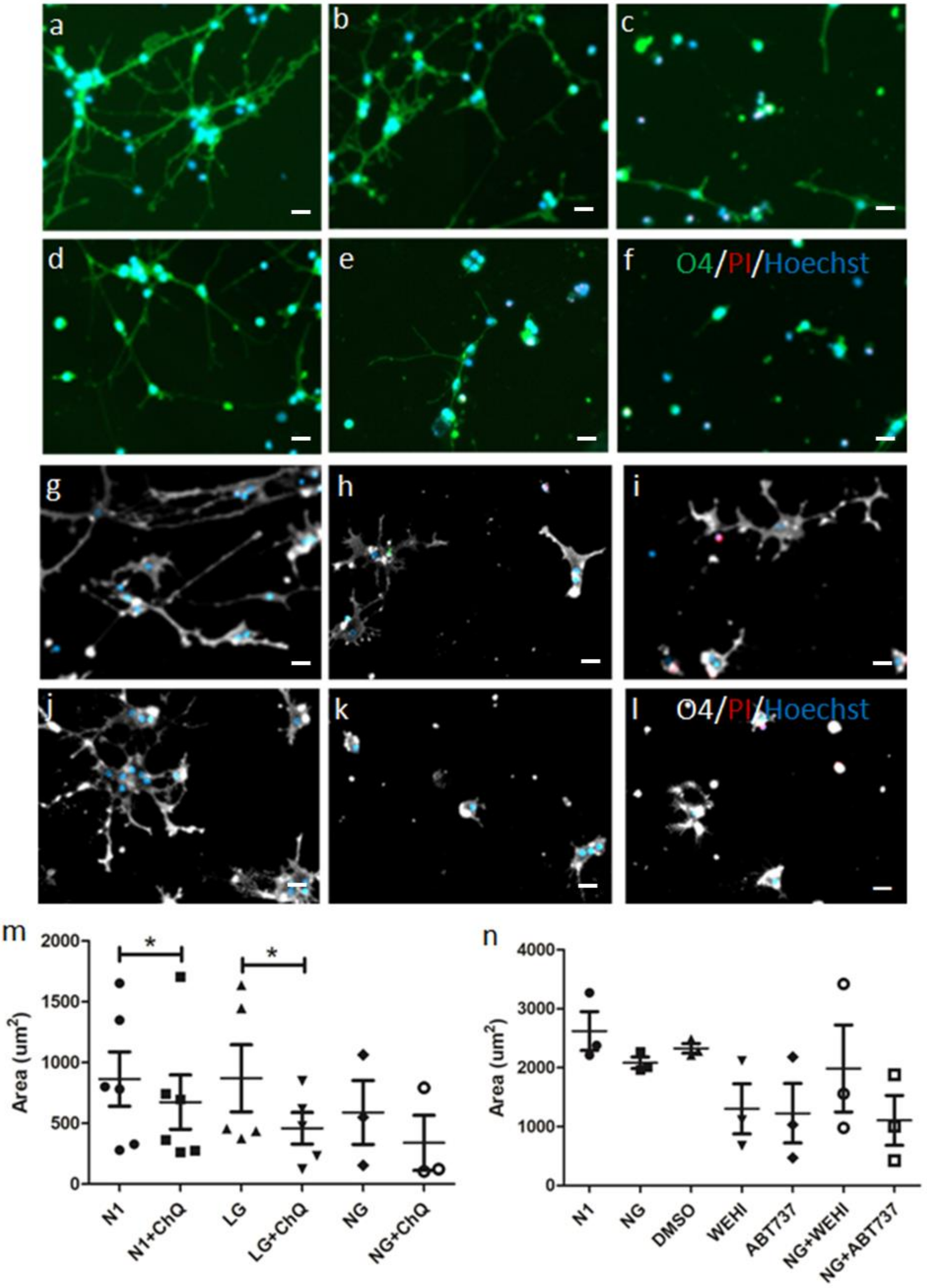
a
b
c
d
e
f
O4/PI/Hoechst
g
h
i
j
k
l
O4/PI/Hoechst
m
Area (um2)
2000
1500
1000
500
0
*
*
N1
N1+ChQ
LG
LG+ChQ
NG
NG+ChQ
n
Area (um2)
4000
3000
2000
1000
0
N1
NG
DMSO
WEHI
ABT737
NG+WEHI
NG+ABT737

Supplementary Figure 6 – Illustrations of changes in process extension by adult human OLs exposed to chloroquine or BCL-2 inhibitors under LG conditions for 2 days. a, g) N1 condition; b) LG condition; c, j) NG condition ;d) N1 condition + chloroquine ; e) LG condition + chloroquine ; f) NG condition + chloroquine ; h) N1 condition + BCL-2 inhibitor WEHI; i) N1 + BCL-2 inhibitor ABT737 ; k) NG conditions + BCL-2 inhibitor WEHI, l) NG condition + BCL-2 inhibitor ABT737 ; m) summary the effects of chloroquine on cell area under N1, LG or NG conditions; n) summary the effects of BCL-2 inhibitors WEHI or ABT737 on cell area under N1 or NG conditions. Each dot correspond to an independent biological samples. Mean±SEM for each condition in the figure. Statistical significance was verified by ANOVA/Tukey test Compared to corresponding control conditions: * $p < 0.05$. Scale bar = 20 μm is shown in l), all other figures are in the same scale.

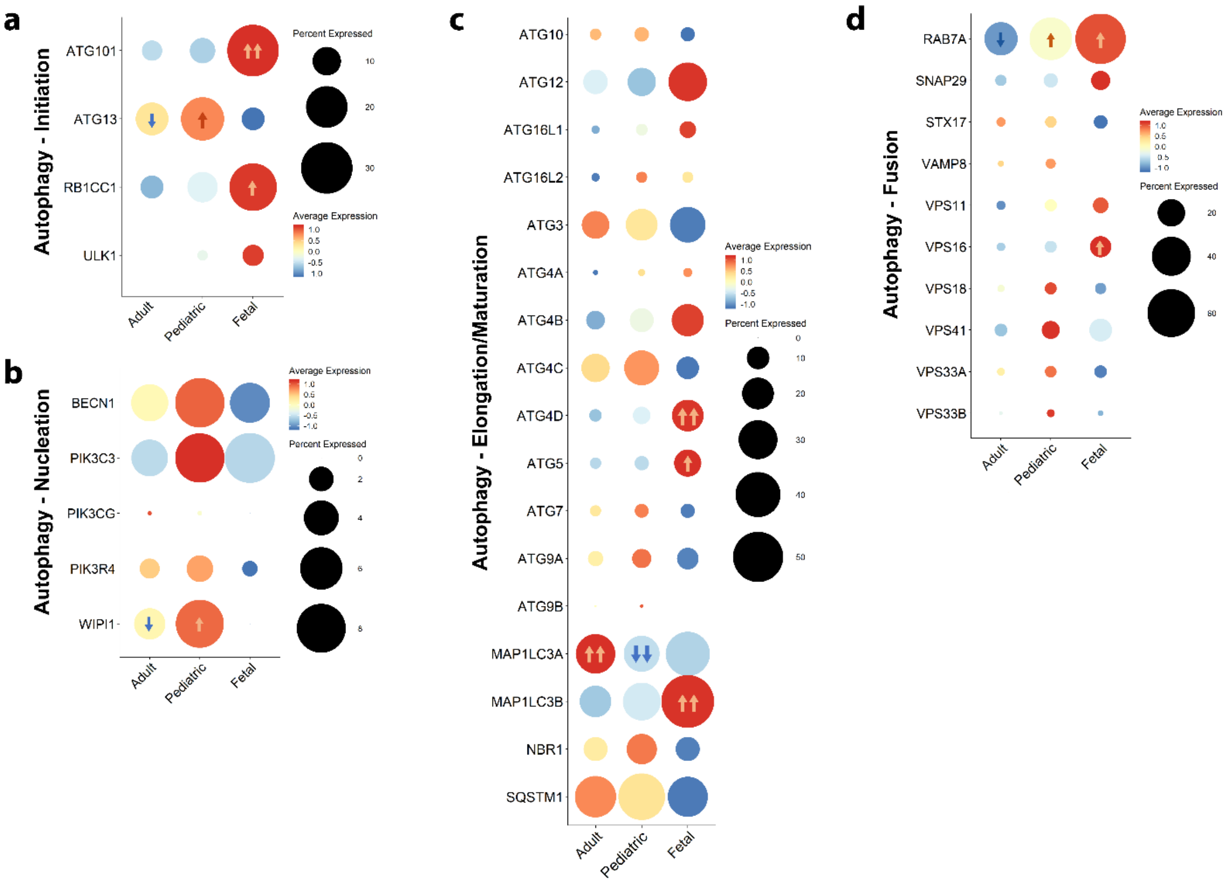

Supplementary Figure 7 – Relative expression of genes related to autophagy pathways. Expression level of genes related to autophagy (**a**) initiation, (**b**) nucleation, (**c**) elongation/maturation, and (**d**) fusion in total OL lineage cells of pooled adult, pediatric, and fetal samples. Scale is z-scores of averaged normalized gene expression across the age groups; percent expressed indicates the percentage of OL-lineage cells in the age group expressing the gene. Arrows indicate up- (orange) or down- (blue) regulation (adj. $p<0.05$) against all other groups, where one arrow $|logFC| > 0.1$, two arrows $|logFC| > 0.25$, by Wilcoxon rank sum differential expression testing with Bonferroni correction. Number of independent biological samples by age group: 4 adult, 7 pediatric and 4 fetal.

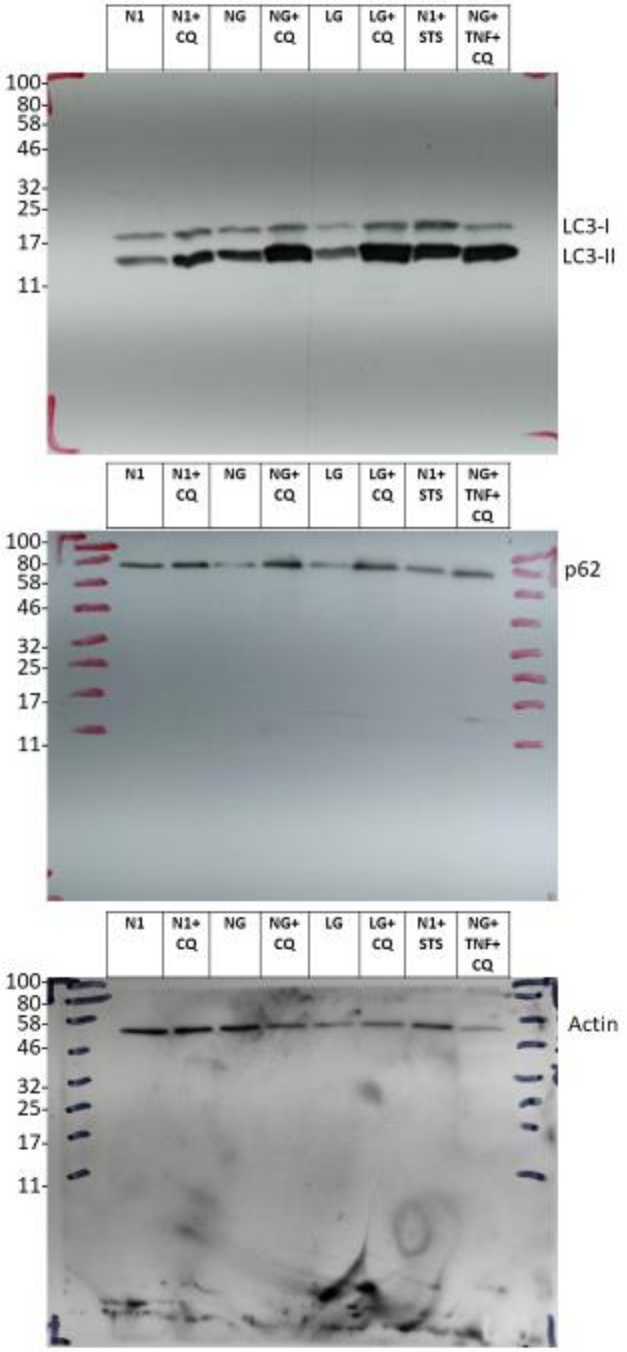

Supplementary Figure 8 – Uncropped blot images for LC3, p62 and Actin (loading control) measurement in the first human oligodendrocyte samples from Figure 6. Molecular weight is indicated at the left of each band and the corresponding protein on the right. Conditions of each lane is indicated at the top of each blot. The last two conditions on the right were not used for this study. All blots are derived from the same gel.

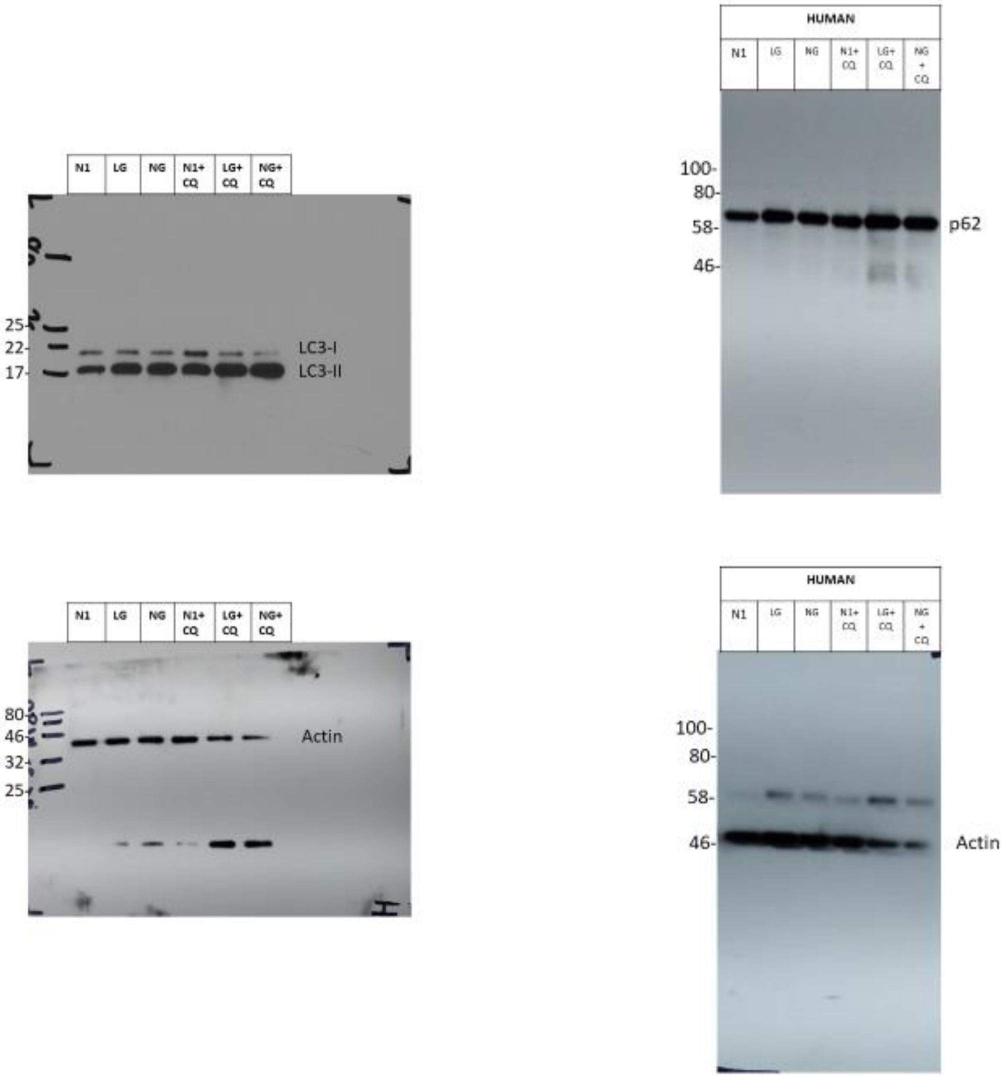

Supplementary Figure 9 – Uncropped blot images for LC3, p62 and Actin (loading control) measurement in the second human oligodendrocyte samples from Figure 6. Molecular weight is indicated at the left of each band and the corresponding protein on the right. Conditions of each lane is indicated at the top of each blot.

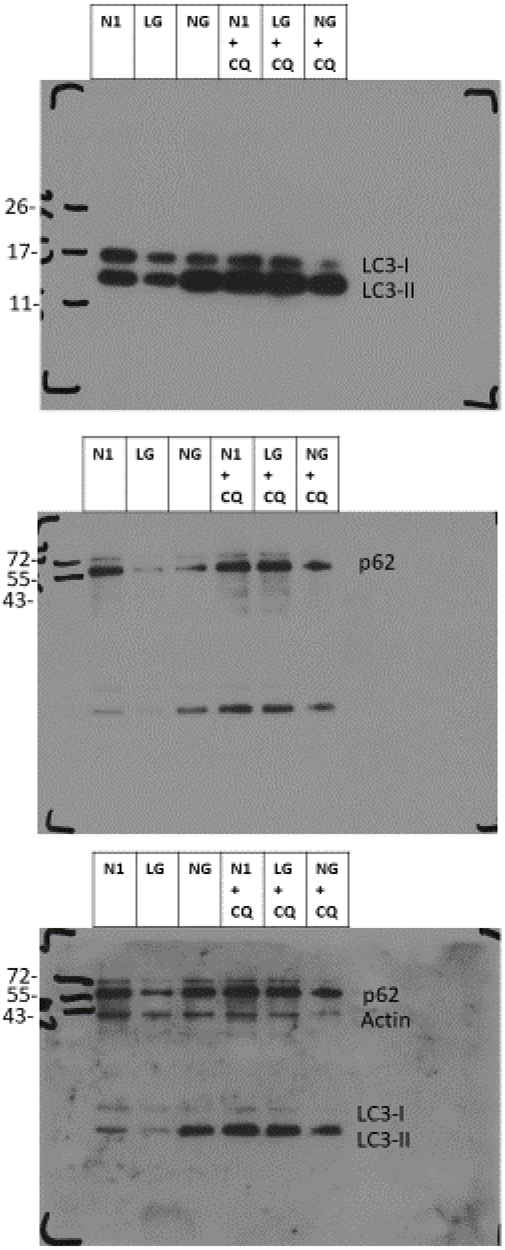

Supplementary Figure 10 – Uncropped blot images for LC3, p62 and Actin (loading control) measurement in the third human oligodendrocyte sample from Figure 6. Molecular weight is indicated at the left of each band and the corresponding protein on the right. Conditions of each lane is indicated at the top of each blot. All blots are derived from the same gel.

Supplementary Table 1 – Pediatric Patient Demographics

	Age	Sex	Diagnosis	scRNAseq	*in vitro* assays
< 5 years	1.5	M	focal cortical dysplasia	+	+
	2	F	focal cortical dysplasia		+
	2	F	megalencephaly	+	
	2	M	Rasmussen's encephalitis	+	+
	4	F	focal encephalomalacia		+
	4	M	focal cortical dysplasia	+	+
	5	M	focal cortical dysplasia		+
> 5 years	7	M	focal cortical dysplasia		+
	8	F	focal cortical dysplasia		+
	10	F	focal epilepsy	+	
	13	F	focal epilepsy	+	+
	14	F	congenital stroke	+	
