# Supplementary material for: Age-related injury responses of human oligodendrocytes to metabolic insults: link to BCL-2 and autophagy pathways
Source: Commun Biol. 2021 Jan 4;4:20. doi: 10.1038/s42003-020-01557-1 (PMC7782481; doi:10.1038/s42003-020-01557-1)
Supplement: Supplementary file 2 — Description of Additional Supplementary Files [file 42003_2020_1557_MOESM2_ESM.pdf]

Description of Additional Supplementary Files

File Name: Supplementary Data 1
Description: Supplementary data provide the original values used to derive the graphs presented in Figures 1 -6.
